# Near Miss in Intraoperative Magnetic Resonance Imaging: A Case for In Situ Simulation

**DOI:** 10.1097/pq9.0000000000000222

**Published:** 2019-09-27

**Authors:** Asheen Rama, Lynda J. Knight, Marc Berg, Michael Chen, Ralph Gonzales, Timothy Delhagen, Lucas Copperman, Thomas J. Caruso

**Affiliations:** From the * Department of Anesthesiology, Perioperative, and Pain Medicine, Stanford University School of Medicine, Stanford, CA; † Lucile Packard Children's Hospital Stanford, Palo Alto, CA; ‡ Department of Pediatrics, Stanford University School of Medicine, Stanford, CA

## Abstract

Pediatric patients in intraoperative magnetic resonance imaging (iMRI) settings are at high risk for morbidity should an adverse event occur. We describe an experience in the iMRI scanner where no harm occurred, yet revealed an opportunity to improve the safety of patients utilizing the iMRI. The perioperative quality improvement team, resuscitation team, and radiology nurse leadership collaborated to understand the process better through in situ simulation.

**Methods:**

After a problem analysis, the team planned an in situ, high-fidelity simulation with predefined learning objectives to identify previously overlooked opportunities for improvement. The iMRI simulation had unique considerations, including the use of a magnetic resonance imaging (MRI)-compatible mannequin and ensuring participants' safety. Audiovisual equipment was placed in strategic locations to record the MRI and operating room (OR) segments of the simulation, and trained health-care simulation experts provided debriefing.

**Results:**

After completion of the iMRI simulation, the quality improvement team solicited feedback from participants and reviewed the video-recorded simulation. Several opportunities for improvement surrounding staff responsibilities and unique aspects of the iMRI environment were identified.

**Conclusions:**

iMRI in situ simulation has not been previously described. It presents unique challenges given the integration of personnel from OR and radiology environments, anesthetized patients, and risks from the high-powered MRI magnet. Other institutions utilizing hybrid ORs with iMRI may consider conducting in situ simulations using the described methods.

## INTRODUCTION

The magnetic resonance imaging (MRI) environment poses a significant danger to patients, and adverse events have been reported, including the death of a 6-year-old after head trauma from a projectile oxygen tank.^1^ Intraoperative MRI (iMRI) is a unique subset of MRIs in which the MRI is performed in conjunction with surgical intervention. The most common application for iMRI is in neurosurgery because it allows surgeons to check for completeness of surgical resection or alter an intraoperative course (such as in stereotactic radiofrequency ablation).^2^ However, the risk for adverse events is increased because of the combination of 2 complex environments—the MRI environment with its high-powered magnet and the operating room (OR) environment with its sterile surgical field and anesthetized patient. Anecdotal correspondences suggest that adverse events and patient safety events in the iMRI environment are not rare. However, very few institutions share their data. To the authors' knowledge, the only published report of an iMRI patient safety event was the description of a surgical retractor that entered the MRI environment.^3^

We experienced an iMRI patient safety event at our institution. Although the event resulted in no harm, it could have resulted in considerable injury. Further, the occurrence itself was indicative of an unreliable process for ensuring the use of nonferromagnetic equipment during procedures. Given this risk, the perioperative quality improvement team, institutional resuscitation team, and radiology nurse leadership collaborated to identify opportunities for process improvement through in situ simulation.

The purpose of this safety report is to highlight a serious event and describe how we undertook simulation-based corrective actions following a root cause analysis. The iMRI in situ simulation served as a platform to uncover latent safety threats and provide staff education.

## METHODS

### Context

The iMRI patient safety event, subsequent root cause analysis, and simulation were performed at Lucile Packard Children's Hospital Stanford, a freestanding, 365-bed academic, pediatric hospital in Northern California. The iMRI scanner and 2 adjoining ORs are staffed by anesthesiologists, surgeons, medical trainees, MRI nurses, MRI technicians, OR scrub technicians, and OR nurses. As a quaternary care trauma center with neonatal, pediatric, and cardiovascular intensive care units, the patient population served in the iMRI and adjoining ORs ranges from complex neonates to adolescents. All nurses and anesthesiologists are required to have advanced life support certifications.

### Event

**Fig. 1.**
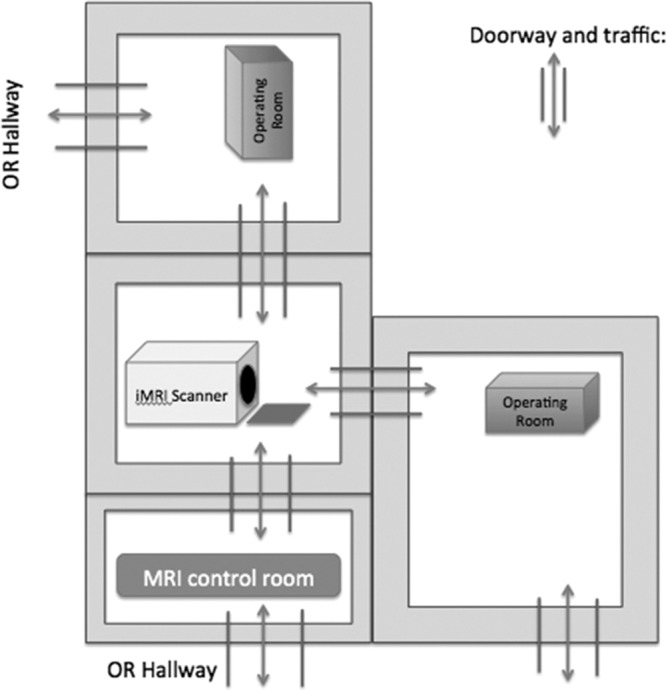
Diagram of iMRI relationship to ORs.

A pediatric patient with refractory epilepsy presented for MRI-guided laser interstitial thermal therapy. The patient was induced under general anesthesia and placed in a prone position. After the head was stabilized in skull pins, the patient was transported from the OR to the adjacent iMRI scanner which housed a stationary magnet (Fig. 1). During the scan, a substantial artifact was noted compared with a typical MRI. We initially attributed the artifact to a scanner malfunction but quickly determined it to be from ferrous metal skull pins. The patient was removed from the scanner and was repinned using the appropriate crystal pins. Although there was a considerable concern for patient burn or projectile injury, no harm occurred, and we completed the scan and surgery without incident.

### Causal Analysis

**Fig. 2.**
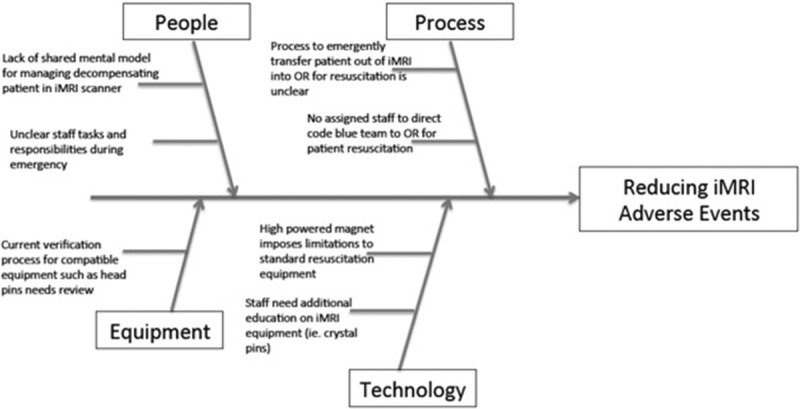
Cause and effect diagram.

Our institution's iMRI surgeries begin with a preoperative time-out during which time the surgeon outlines the positioning and head pinning of the patient. Before the MRI scanner transfer, a multidisciplinary time-out takes place which includes a head to toe assessment for nonferromagnetic MRI equipment and monitors in addition to adherence to a checklist for ensuring the removal of various metal equipment from the patient. The patient is appropriately padded to avoid burn injuries, and auditory protection is applied. The MRI technologist then screens the patient with a handheld metal detector. Our patient safety event occurred because the screening process did not detect the metal headpins. We postulated that the metal detector was not placed close enough in proximity to the patient's head for sterility concerns. This event led to a multidisciplinary root cause analysis with representatives from the perioperative improvement team, institutional resuscitation team, and radiology nurses and technicians. The analysis revealed multiple opportunities in the domains of people, process, equipment, and technology (Fig. 2). Although this particular patient did not require emergent resuscitation, the staff expressed concerns about the lack of a shared mental model on the management of a decompensating patient in the iMRI scanner. During a crisis, the staff did not mutually understand the process for emergently transferring a patient back to the OR from the MRI scanner. Individual responsibilities of the technicians, nurses, and anesthesiologist during an emergency were not well defined, including the process for confirming MRI-compatible surgical equipment

### Intervention

After the initial problem analysis, the team discussed the challenges to realistically simulating an MRI environment given equipment limitations imposed by a magnet and decided to plan an in situ, high-fidelity simulation. The purpose of the simulation was to identify opportunities for improvement and as an educational tool for those present. Based on the problem analysis, the following predefined learning objectives were determined: (1) understanding the process for identifying MRI-compatible surgical equipment, (2) recognizing the clinical deterioration of a patient in the iMRI scanner, (3) demonstrating the initial steps for escalating care when in the iMRI scanner, and (4) demonstrating the most efficient path of travel out of the iMRI scanner.

The scenario involved an anesthetized, neurosurgical patient scheduled for an iMRI surgery starting during the MRI phase of the surgery. As the scenario progressed, the patient became acutely hypoxemic. We chose this scenario to assess the iMRI care team's response to an emergency, including the decision-making process around when to transfer a patient from the scanner back to the OR for safer resuscitation. The team held several meetings before the simulation to ensure staff safety, ultimately resulting in a mock simulation to identify at-risk transitions and the optimal location for audiovisual equipment. Lead OR coordinators ensured the iMRI scanner would be available on the day of the simulation when no elective neurosurgical patients were scheduled for surgeries. On the day of the simulation, staff participating in the simulation were screened for metal using standard MRI protocols. We used a nonferrous, MRI-compatible, pediatric mannequin (Lifecast Body Simulation, Echo Healthcare, Sarasota, FL, USA) for the simulation. A dedicated MRI technologist was responsible for ensuring all participants adhered to MRI safety protocols. A simulation technician positioned cameras, microphones, and simulation monitors in strategic locations to record the entire scenario for postsimulation review without exposing the equipment to the MRI magnet. During the MRI portion of the simulation, cameras in the adjacent MRI control room were used, which were then switched to mounted cameras and microphones in the OR after the mannequin was transported to the adjacent OR.

To provide high-fidelity, in situ simulation, we place MRI-compatible monitoring equipment, including electrocardiogram, pulse oximetry, noninvasive blood pressure cuff, and an arterial line on the mannequin. Vital signs were displayed on a monitor in the adjacent control room during the MRI phase of the simulation. When the simulation participants transported the patient to the OR, the patient's vital signs were transferred to a simulation monitor in the OR. Two MRI technicians, an anesthesiology resident, anesthesiology attending, and a radiology nurse, participated in the 30-minute simulation. Following the simulation, 3 health-care simulation experts led the participants in a guided 20-minute debrief, discussing learning objectives and reflecting on specific educational points.

## RESULTS

After completion of the iMRI simulation, the quality improvement team solicited feedback from the participants and reviewed the recorded simulation. Several key issues surrounding staff responsibilities and unique aspects of the iMRI environment were uncovered, including the process for preventing code team respondents from entering the MRI scanner and the lack of shared criteria for urgent patient removal from the scanner. To provide ongoing education and support for continuous process improvement, we plan future in situ simulations to prevent cognitive drift and review learning objectives.

## DISCUSSION

Although simulation has been used to prepare for iMRI procedures in the adult setting,^4^ to the authors' knowledge, this is the first description of the utilization of a pediatric in situ simulation for iMRI training. As a hybrid environment, iMRI has numerous safety concerns, including the complex care of a neurosurgical patient, and unique safety considerations to MRI-compatible equipment, monitoring, and surgical devices.^5^ With the introduction of larger, multidisciplinary care teams, safety training with defined workflow patterns are necessary for safe patient care.^6^ Simulation has been used to improve patient outcomes through experiential learning and can enhance team performance by improving communication and technical skills.^7^ Also, in situ simulation has been used to train perioperative OR teams,^8^ and in MRI environments to assess team readiness for low-incidence, high-morbidity events.^9^ However, iMRI is a distinct environment that includes both radiology and intraoperative teams. Due to the high-powered magnet located in the hybrid OR, perioperative staff who are unfamiliar with the MRI safety protocols may place themselves and patients at risk. To address this concern, it is important to effectively train dedicated staff before caring for patients in this environment.^3^

However, in situ iMRI simulation also presents challenges, requiring detailed planning. The team held several meetings before the simulation to ensure staff safety, ultimately resulting in a mock simulation to identify at-risk transitions and the optimal location for audiovisual equipment. On the day of the simulation, the team included a dedicated MRI technologist who was responsible for ensuring all participants adhered to MRI safety protocols. Lead OR coordinators ensured the iMRI scanner would be available on the day of the simulation without interruption to patient care.

We present a patient safety event which prompted a unique in situ simulation in a hybrid OR environment. This simulation provided an educational opportunity to our staff and revealed challenges associated with iMRI. Also, the simulation served as a mechanism for staff to share discoverable safety threats in a safe, nonjudgmental environment. Given the identified gaps in communication and roles, the iMRI team conducts a preoperative time-out and another separate time-out before entering the MRI scanner, followed by adherence to a thorough safety checklist. Also, the OR nurse reliably reviews patient implants and potential instruments that would remain on the patient during scans with the radiology technologist. Other institutions utilizing hybrid ORs may consider conducting in situ simulations using the described methods. These simulations can serve to uncover latent safety threats and inform a root cause analysis process.

## DISCLOSURE

The authors have no financial interest to declare in relation to the content of this article.
